# Anti‐MDA5 and anti‐SSA/Ro52 antibodies double‐positive dermatomyositis overlapping with rheumatoid arthritis‐associated interstitial lung disease: A case report

**DOI:** 10.1111/1756-185X.14430

**Published:** 2022-08-27

**Authors:** Hong Xiong, QianRen Tan, Feng Luo, XueMei Yuan, Wukai Ma, XueMing Yao

**Affiliations:** ^1^ Graduate School, Guizhou University of Traditional Chinese Medicine Guiyang China; ^2^ Rheumatology Unit The Second Affiliated Hospital of Guizhou University of Traditional Chinese Medicine Guiyang China

**Keywords:** anti‐melanoma differentiation‐associated 5 gene antibody, anti‐SSA/Ro52 antibody, dermatomyositis, interstitial lung disease, rheumatoid arthritis

## Abstract

Dermatomyositis (DM) is a poorly prognostic autoimmune disease the pathogenesis of which is multifactorial and not clearly defined. DM may be influenced by genes, environment, and immunity. The typical manifestations of DM are Gottron rash, heliotrope rash, rash on the shoulders and buttocks, erythema around fingernails, excessive keratosis of the epidermis, mechanic's hands, and interstitial lung disease (ILD), among others. Anti‐melanoma differentiation‐associated 5 gene (MDA5) antibody has been strongly associated with DM. Furthermore, anti‐SSA/Ro52 antibody has been reportedly associated with DM. A 49‐year‐old woman presented with cough, expectoration, and dyspnea. Relevant examinations revealed elevated levels of muscle enzyme, double‐positive anti‐MDA5 and anti‐SSA/Ro52 antibodies, positive rheumatoid factor, and a high titer of anti‐citrullinated protein antibody. DM overlapping rheumatoid arthritis with ILD was confirmed. We suggest the use of glucocorticoids combined with immunosuppressant therapy, supplemented with gastric and liver protection, and recommend the use of intravenous immunoglobulins and rituximab.

## 1 INTRODUCTION

Dermatomyositis (DM) is a heterogeneous autoimmune inflammatory disease affecting the skeletal muscles and skin. Clinical manifestations include symmetrical proximal muscle weakness and typical skin lesions.^1^ Anti‐SSA/Ro52 and anti‐melanoma differentiation‐associated gene 5 (MDA5) antibodies appear to be associated with myositis.^2^ Interstitial lung disease (ILD) is a relatively common complication of DM, often involving the alveolar wall and perialveolar tissues. ILD is characterized by interstitial inflammation and fibrosis of the lungs.^3^ More than 50% of all cases of ILD associated with DM are positive for anti‐MDA5 antibody.^4^ There are few studies and cases of anti‐MDA5 and anti‐SSA/Ro52 antibodies. Here, we report a rare case of anti‐MDA5 and anti‐SSA/Ro52 antibodies double‐positive DM with rheumatoid arthritis (RA)‐associated ILD.

## 2 CASE REPORT

**FIGURE 1:**
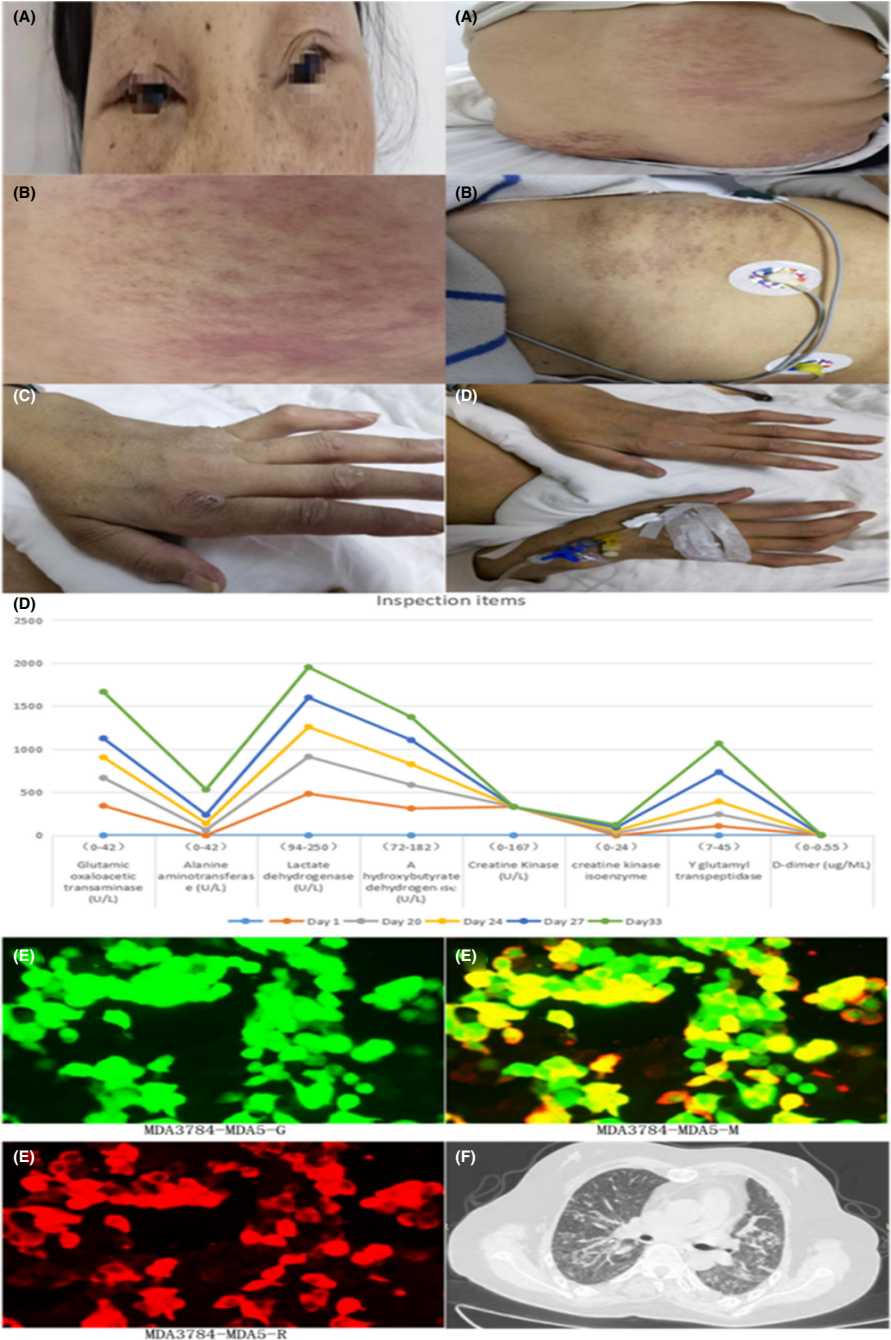
Patient clinical presentation, muscle zymogram, myositis antibody profile and imaging. (A) Typical positivity rash. (B) Typical shawl sign. (C) Gottron sign and joint deformities in rheumatoid arthritis. (D) Changes in muscle zymogram before and after treatment. (E) Positive example of antibody detection by double fluorescent cell transfection (MDA3784‐MDA5‐G, MDA3784‐MDA5‐M, MDA3784‐MDA5‐R) (titer 1:300). (F) Typical cellular shadow in interstitial pneumonia.

A 49‐year‐old woman presented with a 5‐year history of pain in both shoulders, knees, ankles, and especially in both palmar knuckles, with positive rheumatoid factor and high titer of anti‐citrullinated protein antibody, consistent with a diagnosis of RA. The patient presented with cough and expectoration for half a month, with shortness of breath and dyspnea for 4 days. Physical examination revealed purple erythema around both orbits with edema (Figure 1A), large dark red plaques on the back, and scattered rash on the shoulder and left anterior chest, without ulceration or pruritus (Figure 1B). Additional observations were scatter in the skin of both hands, crusting, weakness in the proximal interphalangeal joints of both hands, joint deformation, and gooseneck deformity (Figure 1C). Her lips were slightly cyanotic, the thorax was symmetrical without deformity, respiratory mobility of both lungs was similar, respiratory sounds of both lungs were thick, and dry or wet rales were not heard in either lung. There was no deformity in the spine, redness and tenderness in multiple joints throughout the body, and high local skin temperature. Blood tests showed elevated muscle enzyme (Figure 1D) and positive anti‐MDA5 and anti‐SSA/Ro52 double antibodies (Figure 1E). Chest computed tomography revealed changes consistent with ILD (Figure 1F). Based on these results, we diagnosed double‐positive DM with anti‐MDA5 and anti‐SSA/Ro52 antibodies with RA and ILD.

Treatments included methylprednisolone sodium succinate pulse therapy and oral hydroxychloroquine sulfate tablets to improve the symptoms of DM, oral pirfenidone to improve ILD, intravenous injection of pantoprazole sodium to inhibit acid and protect the stomach, and glycyrrhizin to protect the liver. After treatment with this regimen, the muscle enzyme index of the patient was still high. However, clinical symptoms significantly improved, no new rash developed, and joint pain, weakness, and other symptoms were significantly reduced. It was recommended that the patient receive intravenous immunoglobulins (IVIG) and rituximab (RTX), and that she and her family be discharged due to financial problems. One month later, the patient's family reported the patient's death.

## 3 DISCUSSION

DM is a rare autoimmune disease that mainly affects the skin, muscles, and lung.^5^ Myositis‐specific antibodies are a new class of biomarkers that are valuable in the diagnosis, treatment, and prognosis of diseases. Anti‐MDA5 antibody, formerly known as anti‐CADM140 antibody, was initially identified in patients with clinically amyopathic DM (CADM) and are strongly associated with rapidly progressive ILD.^6^ A strong association has been reported between anti‐Ro52 and anti‐MDA5 antibodies in DM, with a significantly lower survival rate of patients with double‐positive anti‐MDA5 and anti‐Ro52 antibodies than that of patients with only positive anti‐MDA5 antibody.^7^ A North American adolescent myositis cohort study found that 70% of patients with double‐positive DM with anti‐MDA5 and anti‐SSA/RO52 antibodies were diagnosed with ILD, suggesting that anti‐SSA/Ro52 antibody was an independent predictor of ILD.^8^ Compared to DM, RA is a common autoimmune disease. ILD is a serious pulmonary complication of RA, accounting for 10%–20% of the mortality rate, with an average survival time of 5–8 years.^9^ An active marker of anti‐MDA5 antibody‐positive DM–ILD is a concern, and methioninemia is a predictor of poor prognosis.^10^, ^11^ A study in China showed that IVIG treatment reduced ferritin concentration, anti‐MDA5 titer, and lung ground‐glass opacity score, with a significantly lower 6‐mortality rate in the IVIG group than in the non‐IVIG group. However, treatment of ILD related to connective tissue disease involves case reports of patients receiving pirfenidone and nidanib as a definitive treatment. While the treatment was described as effective in improving symptoms, there was a lack of evidence‐based detail.^12^, ^13^ Moreover, DM has a strong association with malignancy, and patients with DM have an increased risk of malignancy at the time of DM diagnosis and 10 years later.

A literature search was performed in PubMed for the last 5 years, using the terms “anti‐MDA5 antibody,” “anti‐SSA/RO52 antibody,” “DM,” “RA,” “ILD,” and “case report”. The search revealed 7 cases of anti‐MDA5 antibody‐positive DM with ILD, 1 case of RA with anti‐MDA5 antibody‐positive ILD, and 2 cases of anti‐MDA5 antibody‐positive DM with systemic lupus erythematosus. Anti‐MDA5 antibody‐positive DM overlapping with other rheumatic diseases is relatively rare, and there are currently no guidelines for the treatment of such diseases. The ideal drug for the treatment of DM is not clear. The treatment plan is mainly based on the results of clinical observation. The current cornerstone of DM therapy is glucocorticoids alone or in combination with an immunosuppressant,^14^, ^15^ among which cyclophosphamide, azathioprine, mycophenolate mofetil, calcineurin inhibitors, RTX combined with corticosteroids are also the choice of most clinicians.^15^, ^16^, ^17^

## 4 CONCLUSION

In this case, 2 autoimmune diseases overlapped, and both led to ILD. The clinical characteristics, laboratory examination data, and immunological characteristics of the patient met both the European Neuro Muscular Centre 2018 DM diagnostic criteria and the 2010 American College of Rheumatology / European Alliance of Associations for Rheumatology RA classification criteria.^18^, ^19^ Thus, the diagnosis was DM–RA overlap syndrome. The patient was previously healthy, and the only underlying disease before DM was RA. The patient complained of irregular medications for RA. As the disease progressed, DM overlapped. The specific cause of DM is still unclear, and more basic and clinical studies are needed to confirm it. The patient died 1 month after discharge from the hospital and failed to complete electromyography and muscle biopsy, which fully explained the rapid progression of the disease. To treat the disease, clinicians must develop a scientific treatment plan, and the patient must cooperate with the treatment to meet the standard procedure.
